# Is fluorometric sentinel lymph node biopsy in endometrial cancer necessary?

**DOI:** 10.3389/fmed.2024.1434311

**Published:** 2024-07-24

**Authors:** Liqiong Huang, Wei Cheng, Chenghui He, Xin Li, Lu Huang, Jiajia Zhang, Liwen Song, Yifan Zhou, Chenchen Wang, Xiaoqin Gan, Jin Qiu

**Affiliations:** ^1^Department of Obstetrics and Gynecology, Shanghai Tongren Hospital, Shanghai Jiaotong University School of Medicine, Shanghai, China; ^2^Department of Obstetrics and Gynecology, Chengdu Women’s and Children’s Central Hospital, University of Electronic Science and Technology of China, Chengdu, Sichuan, China; ^3^Shanghai United Family Pudong Hospital, Shanghai, China

**Keywords:** endometrial cancer, SLNB, case-control study, prognosis, surgery

## Abstract

**Objective:**

In this study, we collected perioperative and postoperative follow-up data from patients with endometrial cancer (EC) at different stages to evaluate the role of sentinel lymph node biopsy (SLNB) in endometrial cancer surgery.

**Methods:**

A total of 186 endometrial cancer patients undergoing radical hysterectomy from January 2018 to April 2022 were retrospectively analyzed. Patients were classified into four groups. Group A comprised patients diagnosed with stage IA grade 1 and 2 endometrioid EC who underwent SLNB. Group B comprised patients with stage IA grade 1 and 2 endometrioid EC who did not undergo SLNB. Group C comprised patients with higher-grade endometrioid EC, wherein systematic lymph node dissection was performed based on SLNB results. Group D comprised patients with higher-grade endometrioid EC who did not undergo SLNB and instead underwent direct systematic lymph node dissection. Clinical, pathological data, and follow-up information for all patients were collected.

**Results:**

In Group A and B, SLNB was performed on 36 out of 67 patients with IA stage 1 and 2 endometrial cancer, yielding a SLN positivity rate of 5.6%. There were no significant differences observed between the two groups regarding perioperative outcomes and postoperative follow-up. Conversely, among 119 patients with higher-grade endometrial cancer, 52 underwent SLNB, with 20 patients exhibiting SLN positivity, resulting in a SLN positivity rate of 38.4%. However, the decision to undergo SLNB did not yield significant differences in perioperative outcomes and postoperative follow-up among these patients.

**Conclusion:**

For stage IA grade 1 and 2 endometrioid EC, the incidence of lymph node positivity is low, omitting SLNB in this subpopulation is a feasible option. In other stages of endometrioid EC, there is no significant difference in perioperative and postoperative follow-up data between patients undergoing routine systematic lymphadenectomy and those undergoing systematic lymphadenectomy based on SLNB results. Therefore, if SLNB is not available, the standard procedure of PLND remains an option to obtain information about lymph node status, despite the surgical complications associated with this procedure.

## Introduction

Endometrial cancer (EC) is the second most common reproductive tract malignancy in China and the first in developed countries (1, 2). According to the National Cancer Centre in 2019, the incidence and mortality rate of EC in China was 0.28/100,000 and 1.9/100,000 respectively, with the incidence rising in recent years (3). Surgical staging with lymphadenectomy can accurately stage EC, helping clinicians perform adjuvant radiotherapy for high-risk patients, ultimately reducing recurrence (4, 5). Patients without lymphadenectomy depend on a preoperative pelvic CT/MRI imaging examination and postoperative pathological examination to determine disease stage. However, the precise EC stage is likely more advanced than that diagnosed by clinicians, and the probability of postoperative supplementary radiochemotherapy and subsequent recurrence is high (6–8).

During surgery for endometrial cancer, dye can be injected into the cervix to perform sentinel lymph node biopsy (SLNB). This technique helps assess the status of pelvic lymph node metastasis, potentially avoiding the need for systematic lymphadenectomy and thereby reducing the incidence of surgical complications (9–12). Current guidelines have provided recommendations regarding the necessity of performing SLNB during EC surgery (13–15). However, due to the high medical standards and multidisciplinary collaboration required for SLNB, many hospitals in China still lack the necessary infrastructure to complete this procedure. In such circumstances, surgeons often opt for direct systematic lymphadenectomy as a substitute for SLNB.

The aim of this study is to evaluate the necessity and role of SLNB in the surgical management of endometrial cancer by collecting perioperative and postoperative follow-up data from patients at various stages of the disease.

## Materials and methods

### Study design and participants

This study is affiliated with the Longitudinal Endometrial Cancer Surgery Study (LoECSS), a comprehensive cohort investigation focusing on minimally invasive gynecological procedures conducted in Chengdu, China (Chinese clinical trial registration number ChiCTR210053483). This study was approved by the Ethics Committee of the Chengdu Women’s and Children’s Central Hospital [No. 2022 (64)]. Written informed consent was obtained from all participants. We retrospectively analyzed the data of EC patients undergoing surgical treatment in our hospital from January 1, 2018, to April 1, 2022. A total of 251 EC patients were hospitalized, among which 186 received surgical treatment. According to the Querleu-Morrow classification (16), if there is no suspicion of cervical tumor infiltration, bilateral salpingo oophorectomy and type A hysterectomy were performed; If there is suspicion of cervical tumor infiltration, bilateral salpingo oophorectomy and type B hysterectomy were performed. All patients were staged according to the 2023 FIGO surgical pathological staging criteria for endometrial cancer (17). Patients were classified into four groups. Group A comprised patients diagnosed with stage IA grade 1 and 2 endometrioid EC who underwent SLNB. Group B comprised patients with stage IA grade 1 and 2 endometrioid EC who did not undergo SLNB. Group C comprised patients with higher-grade EC, wherein systematic lymph node dissection was performed based on SLNB results. Group D comprised patients with higher-grade EC who did not undergo SLNB and instead underwent direct systematic lymph node dissection.

### Diagnosed and graded criteria of EC

EC was diagnosed and graded according to the criteria of FIGO stages (1, 13). The inclusion criteria were as follows: (i) EC patient received surgical treatment; (ii) Patients’ vital signs were stable without contraindications for surgery; (iii) All grade 1 and 2 endometrioid carcinoma patients in stage IA could be considered for the SLNB group; (iv) All endometrial curettage and histopathology (ECHA) confirmed EC by pathology or EC in stage IA with grade 1 and grade 2 endometrioid carcinoma could be considered for hysterectomy, bilateral salpingo-oophorectomy (BSO) without lymphadenectomy.

The exclusion criteria of fluorescent-guided SLNB were as follows: (i) Evidence of >1/2 myometrium infiltration or cervical or extra-uterine disease or high-grade EC (high grade endometrioid, serous, carcinosarcoma and clear cell carcinoma); (ii) Previous hysterectomy or treatment for EC (such as radiotherapy, chemotherapy, or hormonal therapy); (iii) Previous retroperitoneal surgery or lymphadenectomy; (iv) Necessitating neoadjuvant therapy; (v) a history of hepatic impairment or an iodine allergy; (vi) pregnancy.

### Standard operating procedures for EC

All EC patients received da Vinci Xi Surgical System (Intuitive Surgical, Sunnyvale, CA) or transvaginal natural orifice transluminal endoscopic or multiport laparoscopic or transumbilical laparoendoscopic single-site or abdominal surgery. After achieving ascites cells and BSO and developing retroperitoneal spaces, fluorescence-guided SLNB was used to visualize the ICG tracer in the lymphatic tissue using an imaging system with integrated SPY scope near-infrared (NIR) imaging technology (Hd Fluorescent laparoscopic 2,100, Guangdong Opmundi Technology Co., Ltd). A successful mapping was defined by visualization of a channel leading from the cervix directly to at least one candidate lymph node, in at least one hemi-pelvis (10). The time required for SLNs mapping was recorded. The removed SLNs and ascites cells were submitted for intraoperative frozen section examination, followed by routine hematoxylin and eosin (H&E) staining. Patients subsequently underwent hysterectomy. If intraoperative frozen section analysis revealed positive lymph nodes, grossly suspicious non-sentinel lymph nodes (non-SLNs), or unsuccessful pelvic lymph node mapping, complete bilateral pelvic and para-aortic lymphadenectomy (PPALN) was performed in accordance with departmental policy and preoperative informed consent protocols.

For the non-fluorescent tracer EC patients, preoperative ECHA (pathologically confirmed EC) or grade 1 and grade 2 endometrioid carcinoma in stage IA (by hysteroscopic curettage) received hysterectomy, BSO without lymphadenectomy. The patients with stage II and above endometrial cancer received complete pelvic lymphadenectomy with or without periaortic lymphadenectomy, according to the ESGO/ESTRO/ESP guidelines (1, 13).

All surgeries were completed by 11 gynecologists who achieved the ability to do SLNB evaluated through the learning curve. A standard operating procedure for fluorescence-guided SLNB was established to ensure the quality and consistency of the technique.

Indocyanine Green (ICG) tracer (ICG; Dandong Medical innovation Pharmaceutical Co. LTD, Zone B, No.6 yongxiang Street, Donggang City, Dandong City, Liaoning Province) was injected into the cervix after anaesthesia induction. A standard dose of 0.83 mg/mL was created by diluting 10 mL of the stock solution (25 mg/10 mL) into 20 mL of sterile water (18). A 1-mL syringe was used to inject 1 mL of the ICG solution into the uterine cervix at 3 o’clock of the ectocervix to a 1-cm depth, then, the same dosage of ICG solution was injected to the same position to a 0.5-cm depth. A similar procedure was done in the uterine cervix (9 o’clock).

### Data collection

The general characteristics, perioperative outcomes, SLNB characteristics, pathologic characteristics, and postoperative follow-up (including postoperative adjuvant therapy, complications, recurrence, metastasis, and mortality) were compared. Lymph node resection and fluorometric SLNB in different stages of EC were analyzed.

### Statistical analysis

Statistical analysis was performed using SPSS 26.0 (IBM, Armonk, NY, United States). Analysis of variance was used to compare continuous variables, while chi-squared or Fisher’s exact tests were used to compare categorical data. All probability values were checked bilaterally, with *p* < 0.05 regarded as statistical significance.

## Results

Initially, 252 patients diagnosed with EC were recruited in this study. After applying the inclusion and exclusion criteria, 182 patients were included in the final analysis. Of these, Group A comprised 36 cases (19.8%), Group B comprised 31 cases (17.0%), Group C comprised 52 cases (28.6%), and Group D comprised 67 cases (36.8%).

### The role of SLNB in stage IA grade 1 and 2 endometrioid EC patients

There was no significant difference between SLNB and no lymphadenectomy groups in age, body mass index (BMI), hysterectomy type, para-aortic lymphadenectomy, estimated blood loss, hemoglobin (Hb) decrease at postoperative 72 h, VAS score 24 h after operation except for surgical approach and time of flatus after surgery. All pathologic characteristics including histology, grade in endometrioid carcinoma, stage in final pathology, and postoperative follow-up (including radio chemotherapy, complications, recurrence/metastasis and mortality), were not statistically difference. Only more patients in group A received radiotherapy than those in group B (Table 1).

**Table 1:** Clinic-pathological characteristics and postoperative follow-up between SLNB and no lymphadenectomy group.

Variables	Group A (*N* = 36)	Group B (*N* = 31)	*p*
Perioperative characteristics			
Age (year)	52.78 ± 7.54	52.03 ± 8.23	0.700
BMI (Kg/m^2^)	25.47 ± 3.56	24.99 ± 4.03	0.605
Hysterectomy type			0.495
Simple	34(94%)	31(100%)	
Radical	2(6%)	0(0%)	
Para-aortic lymphadenectomy			0.162
Yes	4(11%)	0(0%)	
No	32(89%)	31(100%)	
Estimated blood loss (mL)	74.44 ± 60.87	62.26 ± 73.47	0.460
Hb decrease at postoperative 72 h (g/L)	14.67 ± 8.03	14.86 ± 9.58	0.931
Time of flatus after surgery (h)	27.14 ± 7.52	35.35 ± 10.49	0.001
Pathological characteristics			
Stage (final pathology)			0.361
IA	26(72%)	26(84%)	
IB	6(17%)	3(10%)	
II and above	4(11%)	2(6%)	
Lymph node metastasis			–
Yes	0(0%)	0(0%)	
No	36(100%)	31(100%)	
Postoperative follow-up			
Radiotherapy			0.017
Yes	10(29%)	2(6%)	
No	24(71%)	29(94%)	
Chemotherapy			0.369
Yes	6(18%)	3(10%)	
No	28(82%)	28(90%)	
Complications			0.093
Yes	2(6%)	7(23%)	
No	34(94%)	24(77%)	
Recurrence/metastasis			0.495
Yes	2(6%)	0(0%)	
No	34(94%)	31(100%)	
Mortality			–
Yes	0(0%)	0(0%)	
No	36(100%)	31(100%)	

SLNB, sentinel lymph node biopsy; BMI, body mass index; Hb, hemoglobin; VAS, visual analog pain scores.

### The role of SLNB in higher-grade EC patients

There was no significance between groups C and D in BMI, para-aortic lymphadenectomy, VAS score 24 h, time of flatus after surgery, pathological characteristics, or postoperative follow-up metrics, except for chemotherapy. Age, surgical approach, and hysterectomy type were significantly different; lymph node metastasis and chemotherapy were higher in group D than in group C (Table 2). There is no significant difference in perioperative and postoperative follow-up data between patients undergoing routine systematic lymphadenectomy and those undergoing systematic lymphadenectomy based on SLNB results (Table 3).

**Table 2:** Clinic-pathological characteristics and postoperative follow-up between lymphadenectomy group with or without SLNB (*n* = 119).

Variables	Group C (*N* = 52)	Group D (*N* = 67)	*p*
Perioperative characteristics			
Age (year)	53.46 ± 10.57	54.00 ± 8.34	0.710
BMI (Kg/m^**2**^)	23.58 ± 3.09	23.74 ± 3.28	0.778
Hysterectomy type			0.319
Simple	43(83%)	51(76%)	
Radical	9(17%)	16(24%)	
Para-aortic lymphadenectomy			0.162
Yes	30(58%)	30(45%)	
No	22(42%)	37(55%)	
Hb decrease at postoperative 72 h (g/L)	16.50 ± 6.28	19.27 ± 11.37	0.119
Time of flatus after surgery (h)	31.17 ± 13.93	36.39 ± 14.99	0.085
Pathological characteristics			
Stage (final pathology)			0.265
IA	18(35%)	23(34%)	
IB	9(17%)	11(16%)	
II	8(15%)	7(10%)	
III	15(29%)	23(34%)	
IV	2(4%)	3(4%)	
Lymph node metastasis			0.004
Yes	12(23%)	15(22%)	
No	40(77%)	52(78%)	
Postoperative follow-up			
Radiotherapy			0.068
Yes	18(35%)	31(46%)	
No	30(58%)	25(37%)	
Chemotherapy			0.003
Yes	18(35%)	39(58%)	
No	30(58%)	18(27%)	
Complications			0.242
Yes	12(23%)	22(33%)	
No	40(77%)	45(67%)	
Recurrence/metastasis			0.721
Yes	5(10%)	5(7%)	
No	47(90%)	62(93%)	
Mortality			0.339
Yes	0(0%)	3(4%)	
No	52(100%)	64(96%)	

SLNB, sentinel lymph node biopsy; BMI, body mass index; Hb, hemoglobin; VAS, visual analog pain scores.

**Table 3:** Association between perioperative complications and characteristics.

Variables	Exp(B)	95% CI	*p*
Age (year)	0.921	(0.858,1.258)	0.491
BMI (g/m^2^)	0.925	(0.904,1.038)	0.915
Hysterectomy type	1.610	(1.306,2.241)	0.028
Stage (final pathology)	1.270	(1.054,1.934)	0.032
SLNB	0.942	(0.919,1.015)	0.757
Lymphadenectomy	1.133	(0.943,3.030)	0.112

SLNB, sentinel lymph node biopsy; BMI, body mass index.

Overall, 88 patients received SLNB using indocyanine green, with at least one SLN detected in 78 (89%) patients. Furthermore, 70 patients (80%) had bilateral pelvic or periaortic SLNs detected. The average time from ICG injection to SLN identification was 8.2 min, ranging from 1 to 20 min. The average number of right SLN per patient was 1.4, ranging from 1 to 5, while that on the left was 1.5, ranging from 1 to 3. SLN location was distributed as follows: external iliac (28%), periaortic (20%), internal iliac (19%), common iliac (17%), obturator (14%), presacral (<1%), parametrial (<1%) (Table 4).

**Table 4:** Sentinel lymph node mapping characteristics.

Variables	Total
Time from ICG injection to SLN identification, minuter, Median (interquartile range)	8.3 (4.2–15.5)
Number of right SLN, Median (interquartile range)	2 (1–5)
Number of left SLN, Median (interquartile range)	1 (1–3)
SLN detection rate, n (%)	
Bilateral	70 (80%)
Unilateral	8 (9%)
Failed	10 (11%)
SLN location, n (%)	
External iliac	70 (28%)
Periaortic	50 (20%)
Internal iliac	48 (19%)
Common iliac	42 (17%)
Obturator	34 (14%)
Presacral	2 (<1%)
Parametrial	2 (<1%)

ICG, Indocyanine Green; SLN, sentinel lymph node. Patients, *n* = 88, total SLN, *n* = 248.

The rates of recurrence and metastasis were as follows: five patients occurred in group D, lymph node metastasis (1), pulmonary metastasis (1, moderately differentiated endometrioid carcinoma in stage IA), vaginal stump recurrence accompanied by retroperitoneal metastasis (1), intracranial metastasis (1), vaginal stump recurrence (1, highly differentiated endometrioid carcinoma in stage IB). Two patients occurred in group A: adenocarcinoma of transverse colon (1, highly differentiated endometrioid carcinoma in stage IA). The patients without lymph node dissection (group B) were diagnosed with ECHA or endometrioid carcinoma EC moderately and highly differentiated in stage IA by hysteroscopic curettage. But two cases were diagnosed with a more advanced stage in the final pathological assessment. One case with moderately differentiated endometrioid carcinoma was diagnosed serous EC moderately differentiated in stage II. The other case with ECHA did not receive a preoperative pelvic CT/MRI imaging examination, whose pathology was high differentiated endometrioid carcinoma in IIIA. The death rate across the groups was as follows: three cases occurred in Group D. One case with moderately differentiated endometrioid carcinoma in stage IA died 40 months after surgery, while two cases in stage IVB died 2 months and 14 months after surgery, respectively.

## Discussion

SLNB can be used to reflect the tumors metastasis in the lymphatic drainage area, thereby affecting the surgical scope (19, 20). Nevertheless, because of the high level of medical proficiency and multidisciplinary collaboration required, numerous hospitals in China are still unable to fulfill the conditions necessary for performing this procedure of SLNB. In this study, we collected perioperative and postoperative follow-up data from patients with endometrial cancer at different stages to evaluate the role of SLNB in endometrial cancer surgery.

Previous studies have shown that SLNB plays a certain role in the surgery of EC, which can accurately stage EC, helping clinicians perform adjuvant radiotherapy for high-risk patients, ultimately reducing recurrence (21). The application of SLNB in EC cannot be as sensitive and specific as it is in breast cancer, whose removal of remaining non-sentinel lymph nodes is determined by intraoperative frozen procedures (18, 20).

Previous studies have shown that the risk of positive nodes is only 1.5% in grade 1 IA endometrioid cancer (22, 23), which is consistent with our findings. As the risk of nodal metastases is low or absent in patients with non-invasive endometrioid endometrial cancer, omitting ultrastaging in this subpopulation is also a feasible option (24).

In the study, 19 high-grade EC patients in stage IA (medium risk), underwent PPLAN without positive lymph nodes. Indeed, two retrospective studies demonstrated that a comprehensive lymphadenectomy was associated with overall improved survival among women with intermediate or high-risk EC (25, 26). For high-grade EC in stage IA, PPLAN was recommended for complete surgical staging. Moreover, the SLNB alone is a feasible alternative as per the most recent NCCN and ESGO guidelines (27–29).

Compared to group C, group D had similar pathological characteristics and postoperative follow-up metrics (except for chemotherapy). More EC patients in group D received chemotherapy and showed lymph node metastasis, which confirmed that SLNB did not predominate and did not affect prognosis for high-grade EC in stage IA or EC patients in stage IB or more advanced stages.

This study indicated an in interesting result, which indicated that no SLNB did not seem to affect prognosis but would add the rate of adjuvant therapy in stage IA grade 1 and 2 endometrioid EC patients. Previous studies have shown that although the rate of positive lymph node biopsies is low in low-grade endometrioid carcinoma with <50% myometrial invasion, the occurrence rate of micrometastasis and macrometastasis is approximately 3–4% (30, 31). Conducting SLNB can help detect isolated tumor cells (ITC) and micrometastasis. Despite current debates on whether ITC affects the prognosis of EC patients, ultrastaging remains a relatively better option for patients unless ITC is definitively deemed insignificant.

Previous studies have shown that radiation therapy (RT) and/or chemotherapy (CTX) can be given as adjuvant treatments to prevent the progression of micrometastases (32). For patients with positive lymph node biopsy, postoperative adjuvant therapy can still effectively reduce the recurrence of EC (33). In a study of SLNB in cervical cancer, similar results were found: once lymph node positivity is established, systematic lymphadenectomy does not provide a prognostic advantage to the patient, attributed to adjuvant treatment (13). However, such results should be viewed with caution. Perhaps, eventually, pathological ultra-staging or OSNA is achieved during surgery to improve sensitivity and specificity of intraoperative frozen results, whether to remove remaining non-sentinel lymph nodes could be determined to ensure residual lymph nodes free of tumors. A recent study using the one-step nucleic acid amplification (OSNA) method, that was previously used for intraoperative freezing detection of breast cancer, was used in EC and with improved accuracy and specificity of intraoperative freezing in EC, making it possible to determine whether to clear the remaining non-sentinel lymph nodes (34).

In the present study, at least one SLN was identified in 89% of patients, which is consistent with previous reports of ICG detection in EC (85–100%). Additionally, our bilateral detection rate (80%) is also comparable with other groups (60–97%) (35–37). Currently, ICG is the most commonly used dye for SLNB during endometrial cancer surgery. Our findings, along with previous reports, indicate that intracervical ICG injection with indocyanine green fluorescence imaging has relatively high detection rates in EC (38–40). Moreover, some other tracer agent such as technetium-99 m radiocolloid (Tc-99 m), blue dyes (methylene blue, isosulfan blue and patent blue) were also used as SLNB but with lower overall and bilateral detection rates (41, 42). The required dose of fluorescent tracer used is uncertain, fluctuating between 0.5 and 2.5 mg/mL (43). In our study, the dose of 0.83 mg/mL could clearly detect the SLNs and had efficient mapping.

Our study has several strengths, including a standard operating procedures and continuous postoperative follow-up. In this study, participants were selected using strict inclusion and exclusion criteria. All surgery were performed following the same surgical protocol, ensuring relative consistency in intraoperative information. Additionally, comprehensive data on patients with EC and perioperative outcomes were analyzed, resulting in a relatively complete study design. Importantly, this study included a relatively complete postoperative follow-up. Of the 186 EC patients, three patients died with one patient having moderately differentiated endometrioid carcinoma in stage IA in postoperative 40 months. The patient who died (58-years old) in stage IA received complete surgical staging without postoperative adjuvant therapy and a standardized follow-up. One EC patient (43-years old) with highly differentiated endometrioid carcinoma in stage IA received hysterectomy, BSO and SLNB, had transverse colon metastasis and received re-operation for colectomy. The cases above reminded us that even in the early stages of EC patients, regular postoperative follow-up is necessary for timely detection of metastasis and treatment implementation.

Our study has some limitations: Firstly, our work was limited by its retrospective design. Secondly, the study was performed at a single, large, tertiary referral center. Thirdly, the sample size of fluorescence-guided SLNB and lymphadenectomy was small with a relatively short follow-up time. Fourthly, due to the high cost of molecular analysis, which is not covered by medical insurance in China, only a minority of patients underwent molecular analysis, and therefore, this aspect was not included in our study. Comprehensive understanding of patients’ molecular analysis data would be beneficial for analyzing postoperative treatment outcomes. As the study included a relatively complete postoperative follow-up, there was no significant loss in patient follow-up, thereby ensuring the reliability of the data.

## Conclusion

According to our study, for stage IA grade 1 and 2 endometrioid EC, the incidence of lymph node positivity is low, omitting SLNB in this subpopulation is a feasible option. In other stages of endometrioid EC, there is no significant difference in perioperative and postoperative follow-up data between patients undergoing routine systematic lymphadenectomy and those undergoing systematic lymphadenectomy based on SLNB results. Therefore, if SLNB is not available, the standard procedure of PLND remains an option to obtain information about lymph node status, despite the surgical complications associated with this procedure.

## Data availability statement

The original contributions presented in the study are included in the article/supplementary material, further inquiries can be directed to the corresponding authors.

## Ethics statement

This study was approved by the Institutional Review Board of Chengdu Women and Children’s Central Hospital (No. 2021103) on June 14, 2021. The participants provided their written informed consent to participate in this study.

## Author contributions

LiH: Writing – original draft, Writing – review & editing. WC: Writing – original draft, Writing – review & editing. CH: Writing – original draft, Writing – review & editing. XL: Writing – original draft, Writing – review & editing. LuH: Writing – original draft, Writing – review & editing. JZ: Writing – original draft, Writing – review & editing. LS: Writing – original draft, Writing – review & editing. YZ: Writing – original draft, Writing – review & editing. CW: Writing – original draft, Writing – review & editing. XG: Writing – original draft, Writing – review & editing. JQ: Writing – original draft, Writing – review & editing.
